# Endoscopic resection alone as a potential treatment method for low-risk deep invasive T1 colorectal cancer

**DOI:** 10.1016/j.igie.2023.09.007

**Published:** 2023-09-21

**Authors:** Yuta Kouyama, Shin-ei Kudo, Katsuro Ichimasa, Shingo Matsudaira, Yushi Ogawa, Kenichi Mochizuki, Yuki Takashina, Yuta Sato, Tatsuya Sakurai, Yasuharu Maeda, Hiroki Nakamura, Masashi Misawa, Yuichi Mori, Toyoki Kudo, Takemasa Hayashi, Kunihiko Wakamura, Tetsuo Nemoto, Toshiyuki Baba, Fumio Ishida, Hideyuki Miyachi

**Affiliations:** 1 Digestive Disease Center, Showa University Northern Yokohama Hospital, Yokohama, Japan; 2 Division of Gastroenterology and Hepatology, National University Hospital, Singapore; 3 Clinical Effectiveness Research Group, University of Oslo and Oslo University Hospital, Oslo, Norway; 4 Department of Diagnostic Pathology, Showa University Northern Yokohama Hospital, Yokohama, Kanagawa, Japan

## Abstract

**Background and Aims:**

Although T1b (deep submucosal invasion) is described as an indication of surgical resection with lymph node dissection for T1 colorectal cancer (CRC) in current guidelines, it reportedly has a weaker correlation to the lymph node metastasis (LNM) than other risk factors (lymphovascular invasion, poorly differentiated or mucinous adenocarcinoma, and tumor budding). “Low-risk T1b” CRC, which is defined as a lesion with only T1b and no other risk factors, may not require additional surgical resection. The aim of this study was to evaluate the necessity of surgeries in patients with low-risk T1b CRC from the point of the risk of LNM, recurrence, and long-term outcome.

**Methods:**

From April 2001 to March 2019, a total of 1271 T1 CRCs were resected. Invasion depth and tumor budding were diagnosed according to the Japanese guideline, lymphovascular invasion was evaluated with special staining and immunostaining, and histologic grade was diagnosed by the highest differentiation. A total of 354 patients with low-risk T1b CRC were then analyzed. Among these, 105 patients were resected endoscopically without surgical resection (ER-alone group), and 249 patients were resected surgically (SR group). The rate of LNM, the cumulative recurrence, and overall survival (OS) were examined in each group.

**Results:**

No LNM was observed in either the ER-alone group (0%; 95% CI, 0-4.2) or the SR group (0%; 95% CI, 0-1.8). Five-year cumulative recurrence rate was 0% (95% CI, 0-9) in the ER-alone group and 0% (95% CI, 0%-3%) in the SR group. The 5-year OS rate was 93% (95% CI, 90-96) in the ER-alone group and 95% (95% CI, 93-96) in the SR group (*P* = .35).

**Conclusions:**

ER alone without SR could be a potential treatment method for patients with low-risk T1b CRC.

The implementation of colorectal cancer (CRC) screening has increased the detection of T1 CRC.^1^ With the advances in endoscopic resection (ER), such as EMR, endoscopic submucosal (SM) dissection, and endoscopic full-thickness resection, the proportion of early CRCs amenable to local excision has increased.^2^, ^3^, ^4^ Because of the aging population worldwide and the risk of perioperative adverse events and mortality, minimally invasive treatment is desired.^5^^,^^6^

In T1 CRCs, deep SM invasion (T1b) reportedly signifies malignant characteristics and indicates a higher risk of synchronous lymph node metastasis (LNM).^7^^,^^8^ According to Japanese guideline, lesions with depth of SM invasion ≥1000 μm are defined as T1b CRC and lesions with depth of SM invasion <1000 μm are defined as T1a CRC.^9^ The National Comprehensive Cancer Network guidelines for colon cancer indicate that cases with SM invasion alone can be successfully treated with ER if no other risk factors.^10^ However, CRC guidelines in Europe and Japan and recommendations of the US Multi-Society Task Force on Colorectal Cancer describe T1b as an indication of surgical dissection with lymph node dissection.^9^^,^^11^^,^^12^ However, this concept has recently been questioned by studies including a recent meta-analysis reporting that T1b without other pathologic risk factors (lymphovascular invasion, poorly differentiated or mucinous adenocarcinoma, tumor budding), defined as low-risk T1b CRC, includes a very low risk of LNM and T1b is not an independent risk factor for LNM.^13^, ^14^, ^15^, ^16^, ^17^, ^18^, ^19^, ^20^ Contrary to the rate of LNM in all T1 CRCs (approximately 10%), the risk of LNM is 1.2% to 2.6% in low-risk T1b CRC.^9^^,^^13^^,^^14^^,^^21^, ^22^, ^23^ Therefore, low-risk T1b CRC can be treated endoscopically without additional surgical resection (SR). However, there is no evidence concerning the risk of recurrence and long-term outcome of low-risk T1b CRC after resection.

In the current study, we examined the rate of LNM and the long-term outcomes of low-risk T1b CRC after ER and SR and investigated the necessity of additional SR after ER for low-risk T1b CRC.

## Materials and methods

### Patients

From April 2001 to March 2019, a total of 34,820 colorectal neoplasms excluding advanced CRCs were resected at our institution. T1 CRC was found in 1361 patients, and ER and/or SR with regional lymph node dissection were conducted. No patient received preoperative radiotherapy or neoadjuvant chemotherapy. Patients with other CRCs (n = 32), familial adenomatous polyposis (n = 2), Lynch syndrome (n = 3), inflammatory bowel disease (n = 5), and those who underwent transanal endoscopic microsurgery (n = 5) were excluded. We also excluded patients who had specimens with positive ER vertical margins but who did not undergo additional SR because of the difficulty of precise pathologic diagnosis (n = 20) and those whose surgical specimens were impossible to pathologically evaluate in detail because of tissue damage or loss (n = 21). Two patients who had synchronous resectable liver metastasis and underwent surgery were also excluded (n = 2). Finally, 1271 patients with T1 CRC were included. Among these, 338 harbored lesions with an SM invasion depth of <1000 μm (T1a), and 933 harbored lesions with an invasion depth of ≥1000 μm (T1b). T1b CRCs were further classified according to the risk of LNM: high-risk (those with one or more pathologic risk factors—lymphovascular invasion, tumor budding, or poorly differentiated or mucinous adenocarcinoma histology) or low-risk (those without pathologic risk factors). Data regarding treatment method, age, sex, tumor location, morphologic type, tumor size, SM invasion depth, histologic grade, lymphovascular invasion, tumor budding, metachronous recurrence, and follow-up duration were obtained from the hospital records. Morphology was classified into 3 types according to the Paris endoscopic categorization of superficial neoplastic lesions and Kudo’s morphologic/development classification: flat elevated (0-IIa or laterally spreading tumor); protruded (0-Is, Isp, or Ip); and depressed (0-IIc, IIc + IIa, IIa + IIc, or Is + IIc).^24^, ^25^, ^26^ Patients were divided into 2 groups according to treatment: an ER-alone group (ER alone without additional SR) and a SR group (initial SR or ER with additional SR). All patients provided written informed consent. Hospital ethics committee approval was obtained (approval number: 21-072-B). This study is registered in the University Hospital Medical Network Clinical Trials Registry (UMIN 000046993).

### Endoscopic and surgical procedures

Endoscopic and surgical procedures were performed as previously described.^27^ All colorectal lesions were assessed in real-time using magnifying chromoendoscopy to determine pit pattern classification. Narrow-band imaging and endocytoscopy were also used for diagnostic aids in T1 CRCs. Lesions demonstrating III, IV, or VI low-grade pit patterns (ie, adenomas, intramucosal colorectal carcinomas, and slightly invasive SM colorectal carcinomas) were endoscopically resected. Patients with lesions demonstrating a VI high-grade or VN pit pattern (ie, massively invasive SM colorectal carcinomas) were referred for surgery. Patients with adverse events and/or advanced age, or those who refused surgery, underwent ER as their initial treatment. For all patients with T1b cancers after ER, additional SR was typically recommended; however, some patients did not undergo additional SR due to factors such as advanced age or adverse events.

### Histopathologic evaluation

Two gastroenterology pathologists (T.N. and S.H.) diagnosed all cases. Risk factors for LNM, including depth of SM invasion, histologic type, lymphovascular invasion, and tumor budding, were defined according to the World Health Organization criteria,^28^ the Japanese Society for Cancer of the Colon and Rectum (JSCCR) guidelines,^9^ and the Japanese Classification of Colorectal, Appendiceal, and Anal Carcinoma^29^ guidelines in accordance with a previous report.^30^ Histologic type was diagnosed following the World Health Organization criteria. The depth of SM invasion, lymphovascular invasion, and tumor budding were assessed in accordance with the JSCCR guidelines and the Japanese Classification of Colorectal, Appendiceal, and Anal Carcinoma guidelines. The presence of muscularis mucosae was confirmed using the Desmin antibody (Dako North America Inc, Carpinteria, Calif, USA).

Methods for measuring depth of SM invasion were as follows. When it was possible to identify or estimate the location of the muscularis mucosae, depth of SM invasion was measured from the lower border of the muscularis mucosae. When it was not possible to identify or estimate the location of the muscularis mucosae, depth of SM invasion was measured from the surface of the tumor. For pedunculated lesions with a tangled muscularis mucosae, depth of SM invasion was measured as the distance between the point of deepest invasion and the reference line, which is defined as the boundary between the tumor head and the stalk. Invasion by pedunculated lesions that is limited to within the head is defined as “head invasion.”

Histologic grade was classified according to World Health Organization criteria as follows: well-differentiated adenocarcinoma, moderately differentiated adenocarcinoma, poorly differentiated adenocarcinoma, and mucinous carcinoma. We used the highest differentiation as the representative histology. If a lesion displayed any features of poorly differentiated adenocarcinoma or mucinous carcinoma, it was classified as undifferentiated histology. Therefore, a lesion with predominantly well-differentiated adenocarcinoma and partially poorly or mucinous differentiated adenocarcinoma was handled as poorly or mucinous differentiated adenocarcinoma. Vascular invasion was diagnosed by double staining with hematoxylin and eosin and Victoria blue (Muto Pure Chemicals Co, Ltd, Tokyo, Japan). Vascular invasion was defined as invasion of tumor cells into blood vessels. Lymphatic invasion was diagnosed by hematoxylin and eosin staining and immunostaining with D2-40 antibody (Dako North America Inc). Lymphatic invasion was defined as invasion of tumor cells into lymphatic vessels. Tumor budding was defined as a cancer cell nest consisting of 1 to 5 cells at the invasive margin of the carcinoma.^31^ After selecting the field in which budding was most intensive, the number of buddings was counted with a ×20 objective lens. Budding was graded according to the number of buddings counted: BD1, 0 to 4; BD2, 5 to 9; and BD3, ≥10. According to the JSCCR guidelines,^9^ BD2 to BD3 was defined as tumor budding positive. Surgical specimens were used as the criterion standard for the presence of LNM; we therefore investigated the rates of LNM in the SR group.

### Surveillance after resection

After SR, physical examination and blood testing, including carcinoembryonic antigen and carbohydrate antigen 19-9 levels, were performed (in principle) every 3 months for the first 3 years and then every 6 months for the next 2 years in accordance with the JSCCR guidelines.^9^ In addition, CT imaging of the chest, abdomen, and pelvis was performed every 6 months, and a full colonoscopy was performed every year for 5 years. After ER, physical examination, blood testing (including carcinoembryonic antigen and carbohydrate antigen 19-9 levels), CT imaging of the chest, abdomen, and pelvis and a full colonoscopy were performed every year for 5 years. Diagnosis of recurrence was based on CT imaging, magnetic resonance imaging, colonoscopy, and/or positron emission tomography/CT imaging findings. Local recurrence and distant recurrence were regarded as a recurrence event. Local recurrence was defined as recurrence within the surgical field for colon cancer and recurrence within the pelvis for rectal cancer. Distant recurrence was defined as metastasis of the index tumor. Patients suspected with recurrence were surveyed for other cancers, and those without other cancers were diagnosed as patients with recurrence of CRCs.

### Statistical analysis

Values are reported as means ± standard deviation. Group differences were compared by using the Fisher exact test or Pearson χ^2^ test for categorical variables and the Mann-Whitney *U* test for continuous variables. Cumulative recurrence rate and overall survival (OS) were calculated by using the Kaplan-Meier method and compared by using the log-rank test. All statistical analyses were conducted by using JMP statistical software version 15.0.0 (SAS Institute, Inc, Cary, NC, USA). *P* values <.05 were considered significant.

## Results

### Clinicopathologic characteristics

**Figure 1:**
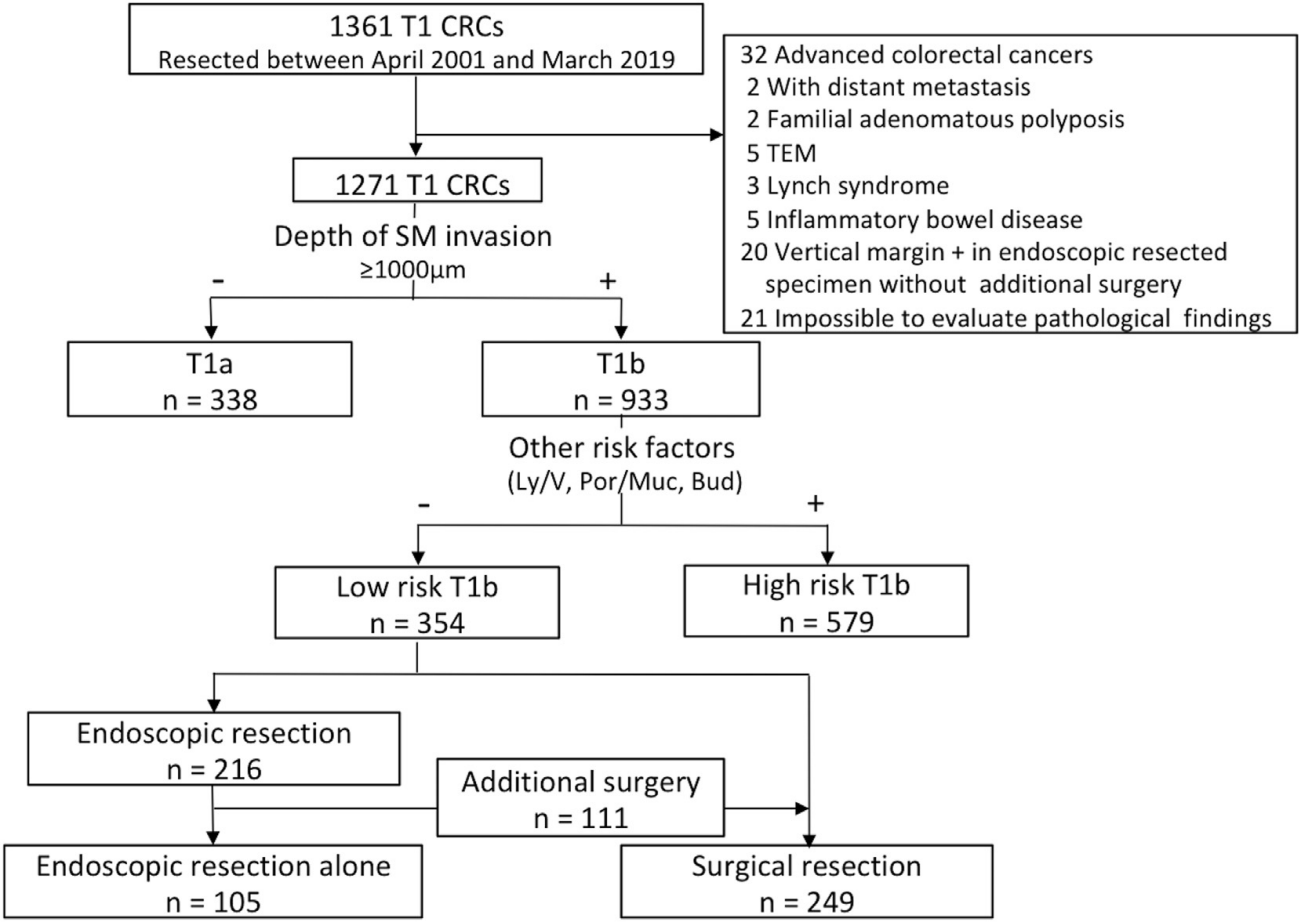
Study flowchart. *CRC*, Colorectal cancer; *TEM*, transanal endoscopic microsurgery; *SM*, submucosal; *Ly/V*, lymphovascular invasion; *Por/Muc*, poorly differentiated or mucinous adenocarcinoma histology; *Bud*, budding grade 2 or 3.

The study flowchart is shown in Figure 1, and characteristics according to the initial treatment are described in Supplementary Table 1, available online at www.igiejournal.org. Among 354 patients with low-risk T1b CRC, 105 cases were in the ER-alone group and 249 cases were in the initial or secondary SR group. Sex, tumor size, and morphologic type did not significantly differ between the ER-alone and SR groups (Table 1). Age and the rate of rectal location were significantly higher in the ER-alone group than in the SR group. The follow-up period was significantly longer in the SR group (54 months in the ER-alone group and 64 months in the SR group).

**Table 1:** Clinicopathologic characteristics of low-risk T1b colorectal cancer in each treatment method

Characteristic	Low-risk T1b		*P* value∗
	ER alone (n = 105)	SR (n = 249)	
Age, y	71 (64.5-77)	67 (59-75)	.007
Sex			.72
Female	43 (41%)	108 (43%)	
Male	62 (59%)	141 (57%)	
Tumor size, mm	19 (14-26)	19 (14-25)	.70
Morphologic type			.20
Flat and depressed	63 (60%)	130 (52%)	
Protruded	42 (40%)	119 (48%)	
Tumor location			.01
Proximal colon	30 (29%)	76 (31%)	
Distal colon	45 (43%)	135 (54%)	
Rectum	30 (29%)	38(15%)	
Initial treatment			<.001
ESD	55 (52%)	18 (7%)	
EMR	34 (32%)	42 (17%)	
EPMR	11 (10%)	27 (11%)	
Polypectomy	5 (5%)	24 (10%)	
Surgical resection	0 (0%)	138 (55%)	
Follow-up, mo	54 (31-83)	64 (52-97)	.006

Values are median (interquartile range) unless otherwise indicated.

*ER*, Endoscopic resection; *SR*, surgical resection; *ESD*, endoscopic submucosal dissection; *EPMR*, endoscopic piecemeal mucosal resection.

∗ To compare characteristics between subgroups, the Fisher exact test or Pearson χ^2^ test was used for categorical variables, and the Mann-Whitney *U* test was used for continuous variables.

### The rate of LNM, recurrence, and OS

**Figure 2:**
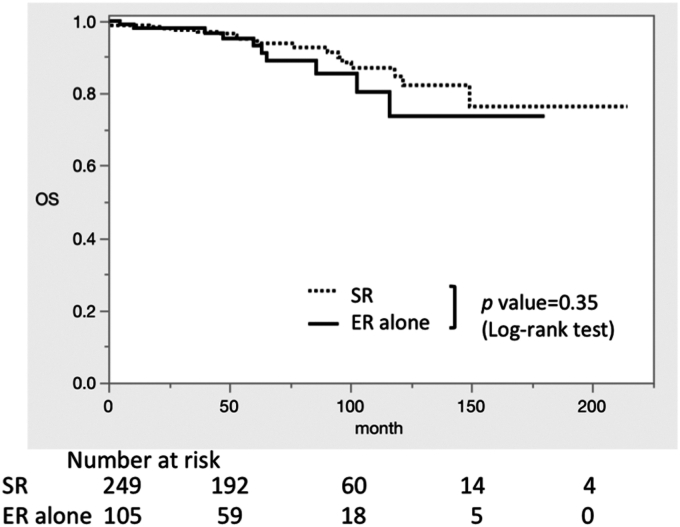
Kaplan-Meier curves of overall survival. *OS*, Overall survival; *SR*, surgical resection; *ER*, endoscopic resection.

Table 2 shows the rate of LNM, 5-year cumulative recurrence, and OS. No LNM was observed among 249 surgically resected T1b CRCs (0%; 95% CI, 0-1.8), and no LNM was observed on subsequent oncologic surveillance among the 105 endoscopically resected T1b CRCs in patients harboring low-risk T1b CRC (0%; 95% CI, 0-4.2). In the SR and ER-alone groups, the cumulative recurrence rate in 5-year was 0% (95% CI, 0-3) and 0% (95% CI, 0-9), respectively, and the 5-year OS rates were 95% (95% CI, 93-96) and 93% (95% CI, 90-96). Figure 2 shows the Kaplan-Meier curve for OS in the SR and ER-alone groups. There was no difference between the SR and ER-alone groups (*P* = .35).

**Table 2:** The rate of lymph node metastasis, 5-year cumulative recurrence, and overall survival of low-risk T1b colorectal cancer in each treatment method

	Low-risk T1b	
	ER alone (n = 105)	SR (n = 249)
Lymph node metastasis	0% (0-4.2)	0% (0-1.8)
5-year recurrence rate	0% (0-9)	0% (0-3)
5-year OS	93% (90-96)	95% (93-96)

Values are rate (95% confidence interval).

*ER*, Endoscopic resection; *SR*, surgical resection; *OS*, overall survival.

## Discussion

In this study, we examined the rate of LNM and the long-term outcomes of patients with low-risk T1b CRCs and investigated the necessity of additional SR with lymph node dissection after ER. Neither LNM nor recurrence was observed in patients with low-risk T1b CRCs, and there was no difference in the OS between the SR and ER-alone group; therefore, ER without additional SR would be a potential treatment approach for patients with low-risk T1b CRC.

Previous studies showed that T1b is not an independent risk factor for LNM, and the risk of LNM is low (2.6%) in T1b CRCs without other histologic risk factors.^13^ The JSCCR guidelines describe the rate of LNM in low-risk T1b CRCs as 1.3% (95% CI, 0-2.4).^9^ The AJCC 8^th^ Edition TNM classification does not subclassify T1 into T1a and T1b because the SM invasion depth does not matter as much as the presence of other high-risk histopathologic features such as lymphovascular invasion, poorly differentiated adenocarcinoma or mucinous adenocarcinoma components (Por/Muc), and high tumor budding.^32^ Concerning the recurrence, previous studies that examined recurrence after resection of T1 CRC have shown that T1b is not an independent risk factor for recurrence.^22^^,^^33^^,^^34^ We expected that T1b alone would not be an important risk factor for either LNM or recurrence. In addition, several studies have reported that SR after ER of T1 CRC does not have an impact on the incidence of adverse events or recurrence.^35^, ^36^, ^37^, ^38^ In our institute, the rate of en bloc resection for all T1b CRC was 84.0% (424 of 505), and complete ER (pathologic vertical margin negative) was 82.4% (416 of 505). The rates of complete ER with endoscopic SM dissection for T1b CRCs reportedly ranged from 64.7% (22 of 34) to 85% (55 of 65) in previous studies.^38^^,^^39^ The rate would not be very low, and ER contains very low risk of lethal adverse events. Therefore, first-line ER accompanied with additional SR based on pathologic findings is an acceptable treatment strategy for all T1 CRCs.^40^ For this treatment strategy, the diagnosis of the cancer as a T1b CRC or invasion into the muscle layer is important. It can be diagnosed with white-light imaging, and the area under the curve was reportedly .80 and .89.^41^^,^^42^

There was no recurrence for low-risk T1b CRCs. However, Ikematsu et al^43^ reported that rectal location is a risk factor for recurrence in low-risk T1 CRCs. We also reported that rectal location is a risk factor for recurrence in high-risk T1 CRCs.^33^ Therefore, careful attention would be needed after ER, especially for rectal cancers.

We also examined the long-term outcomes of low-risk T1b CRC. The 5-year OS rates were 95% and 93% in the SR group and ER-alone group, respectively, and the causes of death were other diseases such as other cancers, pneumonia, cardiovascular disease, or senility. The 5-year OS rate of high-risk T1 CRC after SR was reported as 95.1% and was almost the same as in the ER-alone group (93%).^44^ Therefore, SR for low-risk T1b CRC would not improve the long-term outcome, and ER alone without SR would be acceptable.

However, these results were acquired under strict pathologic evaluations. Special staining (Victoria blue) and immunostaining (D2-40 antibody) were used for the diagnosis of lymphovascular invasion in all T1 CRCs, and the histologic grade was determined by the highest differentiation, and not by the predominant grade.^45^ Among all 933 T1b CRCs in this study, 46 cases only had poorly differentiated adenocarcinoma or were mucinous adenocarcinoma (Por/Muc) positive in the highest differentiation method. In addition, among the 46 cases, 1 case had LNM. In the predominant differentiation method, no case was only Por/Muc, and all cases had other pathologic risk factors (ie, lymphatic or vascular invasion, tumor budding). Therefore, 46 cases can be reduced using the predominant grade method, although 1 case contained LNM. In addition, we reviewed the rate of lymphovascular invasion and Por/Muc components in previous studies (Supplementary Table 2, available online at www.igiejournal.org).^22^^,^^46^, ^47^, ^48^ The rate of lymphovascular invasion and Por/Muc were different in each study. The range of lymphovascular invasion was 12% to 42% and that of Por/Muc was 3% to 13%. The rates of these factors were higher in this study than in other studies. These strict pathologic evaluations would contribute to prevent overlooking of necessary SRs. A previous study identified moderately differentiated adenocarcinoma as a risk factor for LNM, but it used predominant histology as the histologic grade.^49^ Therefore, some cases of moderately differentiated adenocarcinoma may include poorly differentiated or mucinous adenocarcinoma. In the near future, we need to determine whether predominant histology or the highest degree of differentiation is the superior diagnostic criterion for predicting LNM.

In this study, among 354 patients with low-risk T1b CRC, 105 patients (30%) did not undergo SR, possibly because of advanced age and/or adverse events; the median age was higher in the ER-alone group than in the SR group in this study. In previous studies, 24% to 28% of patients with a risk of LNM underwent ER alone, and thus the rate in this study would be normal.^50^^,^^51^

This study has some potential limitations. First, it was conducted in a single institution; consequently, there could be regional or institutional selection biases, and thus we need to conduct a multicenter study in the near future. However, single-center studies also have strengths, including consistency in therapeutic procedures, pathologic diagnosis, and methods of surveillance.^52^ Second, this study was retrospective in nature and was not randomized; therefore, some selection biases may exist. Some patients with adverse events and/or old age were followed up without SR after ER . Further investigation (eg, a prospective randomized study) would be needed. Third, 2 gastroenterology pathologists conducted the pathologic diagnosis; therefore, some differences probably exist in diagnosis. In the future, artificial intelligence–based diagnosis can overcome the low kappa value of diagnosis in pathologic factors.^53^

In conclusion, ER without additional SR would be a potential treatment approach for patients with low-risk T1b CRC, as neither LNM nor recurrence occurred in this study, and there was no difference in long-term outcomes between SR and ER alone in low-risk T1b CRC cases.

## Disclosure

All authors disclosed no financial relationships.

## Figures and Tables

**Supplementary Table 1:** Characteristics of pT1b colorectal cancers by the initial treatment method

Characteristic	Initial ER (n = 216)	Initial SR (n = 138)	*P* value∗
Age, y	68 (59-75)	69 (60-75)	.67
Sex			1.00
Female	92 (43%)	59 (43%)	
Male	124 (57%)	79 (57%)	
Tumor size, mm	18 (14-25)	20 (15-25)	.35
Morphologic type			<.001
Depressed	16 (7%)	50 (36%)	
Flat	86 (40%)	41 (30%)	
Protruded	114 (53%)	47 (34%)	
Tumor location			.25
Proximal colon	58 (27%)	48 (35%)	
Distal colon	113 (52%)	67 (49%)	
Rectum	45 (21%)	23 (17%)	
Pit pattern			<.001
V_I_ low grade	84 (39%)	16 (12%)	
V_I_ high grade	91 (42%)	64 (46%)	
V_N_	9 (4%)	58 (42%)	
Others	32 (15%)	0 (0%)	

Values are median (interquartile range) unless otherwise indicated.

*ER*, Endoscopic resection; *SR*, surgical resection.

∗ To compare characteristics between subgroups, the Fisher exact test or Pearson χ^2^ test was used for categorical variables, and the Mann-Whitney *U* test was used for continuous variables.

**Supplementary Table 2:** The positive rate of lymphovascular invasion and poorly differentiated or mucinous adenocarcinoma in T1 colorectal cancer

Author/year	Ly	Staining∗	V	Staining∗	Ly and/or V	Por/Muc	Grade
Current study	28% (361/1271)	+	27% (337/1271)	+	42% (531/1271)	13% (163/1271)	Highest
						.5% (7/1271)	Predominant
Oh et al, 2019^46^	NA	NA	NA	NA	31% (485/1555)	3% (46/1555)	Predominant
Rönnow et al, 2022^15^	NA	–	NA	–	8% (115/1355)	Por, 8% (105/1348)	Predominant
						Muc, 7% (93/1369)	Predominant
Yasue et al, 2019^54^	NA	±†	NA	±†	42% (359/846)	11% (93/846)	Highest
Barel et al, 2019^48^	22% (64/292)	+	7% (20/292)	+	12% (37/312)	Por, 5% (16/312)	Predominant
						Muc, 7% (22/312)	Predominant
Yoshii et al, 2014^22^	6% (22/388)	NA	7% (26/388)	NA	NA	8% (32/388)	Predominant

*Ly*, Lymphatic invasion; *V*, vascular invasion; *Por*, poorly differentiated adenocarcinoma; *Muc*, mucinous adenocarcinoma; *NA*, not applicable; *+*, special staining and/or immunostaining were performed for diagnosis; *−*, special staining and/or immunostaining was not performed for diagnosis; *±*, special staining and immunostaining were performed for endoscopically resected samples and not for surgically resected samples.

∗ Special staining and/or immunostaining.

† Special staining and immunostaining were performed for endoscopically resected samples and not for surgically resected samples.
